# Improving Knowledge of Needle-Stick Injury Prevention: A Two-Cycle Clinical Audit From Sudan

**DOI:** 10.7759/cureus.96964

**Published:** 2025-11-16

**Authors:** Faris Jamalaldeen Mohammed Hamed, Muhanned Kheder, Osama S Haroon, Abdalla Omer Mohammed Abdelrahman, Marwa Yousif, Islam Yahia Abdalrahman Yagoub, Doaa Elhadi Elimam Mohamed, Eman Ahmed, Shiraz Bashir Jabralseed Mohammed, Wiaam Abdelgadir Msaad Osman, Nejween Abdulaziem Mohammed Ahmed Alawad, Husam Eldin Abuelgassim Hassan Balila, Aia Abdelsalam Elimam Ibrahim, Fahd Elfatih Babiker Elnour, Mogahid Hamdan Adam Ahmed, Hibatallah Mohammed Ali Abass, Eiman AbdElwahab Altayeb, Leena Ibrahim Hassan Mohamed, Elwathig Abdalla

**Affiliations:** 1 Orthopedic Surgery, Bashair University Hospital, Khartoum, SDN; 2 Family Medicine, University of Alberta, Alberta, CAN; 3 General Surgery, Bashair University Hospital, Khartoum, SDN; 4 General Surgery, Managil Teaching Hospital, Managil, SDN; 5 Emergency Medicine, Bashair University Hospital, Khartoum, SDN; 6 Family Medicine, Bashair University Hospital, Khartoum, SDN; 7 Internal Medicine, Bashair University Hospital, Khartoum, SDN; 8 Surgery, Bashair University Hospital, Khartoum, SDN; 9 Orthopedic Surgery, Sudan Medical Specialization Board, Khartoum, SDN

**Keywords:** audit, healthcare workers, needle-stick injury, occupational safety, post-exposure prophylaxis, sudan

## Abstract

Background

Needle-stick injuries (NSIs) remain a major occupational hazard for healthcare workers, exposing them to blood-borne infections. Globally recognized occupational health standards emphasize structured training, safe sharps handling practices, and timely access to post-exposure prophylaxis (PEP). This audit evaluated staff knowledge and awareness of NSI prevention and management in a Sudanese teaching hospital, aiming to identify gaps and assess the impact of targeted interventions.

Methods

A two-cycle clinical audit was conducted over twelve months (September 2024-August 2025) at Bashair University Hospital. A structured, self-administered questionnaire was distributed to doctors, nurses, and laboratory technicians in both cycles (n = 90 per cycle). The tool assessed knowledge of NSI risk pathogens, immediate first-aid response, PEP initiation, sharps disposal practices, vaccination awareness, and reporting procedures. Interventions included structured teaching sessions, posters, departmental reinforcement, and distribution of guideline summaries. Data were analyzed descriptively and compared between cycles.

Results

Knowledge of HBV as the most common pathogen improved substantially among doctors (15.8%→81.2%), nurses (15.8%→100%), and technicians (65%→100%). Awareness of immediate wound washing also improved, reaching 100% among nurses and technicians. Understanding of PEP as medication increased markedly (doctors: 52.6%→93.8%; nurses: 52.6%→100%; technicians: 25%→100%). However, awareness of formal reporting systems declined sharply among doctors (89.5%→12.5%) and technicians (50%→5%). Participation in refresher training fell across groups, while perceived training adequacy showed only partial improvement.

Conclusion

Targeted interventions improved healthcare workers’ knowledge of NSI prevention and management; however, persistent gaps in training sustainability and reporting culture indicate that these gains may not be maintained without continued institutional support. Sustained improvement requires structured refresher programs, robust reporting systems, and administrative commitment to embedding sharps safety into routine practice.

## Introduction

Needle-stick injuries (NSIs) are among the most frequent occupational hazards in healthcare and a major route for transmission of blood-borne pathogens, particularly hepatitis B virus (HBV), hepatitis C virus (HCV), and human immunodeficiency virus (HIV) [1]. The World Health Organization’s modelling estimated that percutaneous exposure to contaminated sharps results in approximately 66,000 HBV infections, 16,000 HCV infections, and 1,000 HIV infections among healthcare workers globally each year, underscoring the substantial occupational risk in routine clinical practice.

Across settings and professions, NSIs remain highly prevalent. Recent syntheses report substantial exposure rates among nurses and physicians worldwide, with a particularly high burden in low- and middle-income regions. For example, a comprehensive meta-analysis published in the Journal of Clinical Nursing found a high global prevalence among nurses. In addition, a systematic review from the Eastern Mediterranean Region (EMR) reported an annual incidence of approximately 43%, while studies from the African Region demonstrated some of the highest exposure rates globally. These figures reflect persistent system-level gaps in prevention, training, and resourcing [2].

Risk is not uniformly distributed: injuries cluster in high-throughput and procedure-dense areas (e.g., perioperative services) and are amplified by modifiable practices such as recapping and suboptimal sharps disposal. Data informed by the International Safety Center’s EPINet indicate that a large share of reported sharps injuries occurs in perioperative care, while multiple studies show that underreporting remains common, ranging from roughly one-third to nearly half of incidents, which delays post-exposure evaluation and prophylaxis [3].

Robust preventive frameworks exist. The Occupational Safety and Health Administration (OSHA)’s Bloodborne Pathogens Standard mandates an exposure control plan, engineering controls (including safety-engineered devices), avoidance of recapping except in limited circumstances, and HBV vaccination, while Centers for Disease Control and Prevention (CDC) resources provide practical, facility-level toolkits to build sharps-safety programs and reduce injuries through training, safer device adoption, and workflow redesign. Up-to-date CDC guidance on blood-borne exposure risks further details immediate management and follow-up after percutaneous injuries [4].

Within the EMR/African context, NSI prevalence and barriers such as inconsistent availability of safety devices, gaps in training, and HBV immunization shortfalls are well-documented, including emerging evidence from Sudan highlighting exposure, frequent recapping, and underreporting. Against this backdrop, our audit at Bashair University Hospital aimed to assess baseline knowledge and awareness of NSI prevention and management among healthcare workers, implement targeted educational interventions to address identified gaps, and subsequently reassess post-intervention improvement in knowledge, reporting practices, and post-exposure management compliance [5].

## Materials and methods

Study design and setting

This clinical audit was conducted at Bashair University Hospital, a tertiary healthcare facility that provides services across multiple specialties, including surgery, obstetrics, pediatrics, internal medicine, emergency medicine, and laboratory services. The hospital environment involves frequent use of needles and sharps, placing healthcare workers at significant occupational risk. The audit was designed according to the classical audit cycle, consisting of a baseline assessment (Cycle 1), identification of knowledge gaps, targeted educational interventions, and a follow-up re-audit (Cycle 2) to evaluate the impact of the interventions. The first cycle was undertaken between September 1, 2024, and February 28, 2025, while the second cycle was conducted between March 1, 2025, and August 31, 2025. The emphasis of this project was on evaluating healthcare workers’ knowledge, awareness, and compliance with safe sharps-handling practices and post-exposure management protocols, rather than on measuring the incidence of NSIs.

Audit standards

Benchmark standards were drawn from authoritative guidance issued by the OSHA and then adapted to the institutional context of Bashair University Hospital [6]. Specifically, engineering and work-practice controls (including the use of safety-engineered devices) and training requirements were based on OSHA’s Bloodborne Pathogens Standard, which requires employers to review technology and implement safer medical devices, prohibit two-handed recapping except in limited circumstances, maintain sharps containers close to the point of use, and provide HBV vaccination and post-exposure evaluation as part of a written exposure control plan [6].

Foundational injection and sharps-safety principles (e.g., standard precautions, safe handling and disposal, and avoidance of needle recapping) were aligned with the WHO’s Best Practices for Injections and Related Procedures Toolkit, which sets the international benchmark for safe injection practices to protect both patients and healthcare workers [7].

Standards for immediate first aid and reporting after an exposure emphasized prompt wound care (washing with soap and water, irrigating mucous membranes or eyes) and urgent evaluation. These elements were anchored in CDC/National Institute for Occupational Safety and Health (NIOSH) guidance on bloodborne exposures and sharps injuries, which specifies immediate washing/irrigation and prompt reporting, and in the United States Public Health Service (USPHS) occupational HIV exposure guidelines that classify occupational exposures as urgent medical concerns, requiring post-exposure prophylaxis (PEP) initiation as soon as possible (preferably within hours, and not later than 72 hours) [8,9].

For post-exposure testing and prophylaxis, we referenced the USPHS occupational HIV PEP guideline (covering risk assessment, PEP regimen and duration, and follow-up testing) and CDC guidance on HCV exposures in healthcare personnel, which recommends baseline and serial testing with initial testing ideally performed within 48 hours. OSHA’s Bloodborne Pathogens Standard also requires the employer to arrange immediate post-exposure evaluation, including source-patient testing where feasible [6,8,9].

Finally, expectations regarding hepatitis B vaccination and documentation of immunity for at-risk staff were aligned with the Advisory Committee on Immunization Practices (ACIP)/CDC recommendations for healthcare personnel (HBV vaccination of HCP and post-vaccination anti-HBs testing when indicated), together with OSHA’s requirement that HBV vaccine be offered within 10 working days of assignment to exposure-risk duties [6,10]. In our audit, we translated these international frameworks into measurable local targets for staff knowledge and awareness. Specifically, we aimed for at least 95% of healthcare workers to have received training on sharps safety within the past 12 months, 100% awareness of the prohibition on needle recapping, at least 95% ability to correctly describe the immediate first-aid and reporting steps following a needle-stick injury, and at least 95% awareness of hepatitis B vaccination requirements, HIV PEP, and follow-up protocols.

Participants and inclusion criteria

The audit population consisted of healthcare workers considered at risk of occupational exposure to sharps. This included doctors, nurses, laboratory personnel, and support staff employed in high-risk clinical areas. A total of 90 participants were enrolled in each audit cycle, comprising 50 doctors, 20 nurses, and 20 laboratory technicians. Staff members who had worked at the hospital for less than three months during the audit periods or who were absent due to extended leave were excluded from the study. Participation was voluntary, and confidentiality was strictly maintained. During both audit cycles, structured questionnaires were distributed to eligible staff, and only fully completed forms were included in the final analysis. No formal power calculation was performed, as the audit aimed to include the entire accessible population of at-risk healthcare workers during the study period; therefore, the sample reflects the complete eligible workforce rather than a statistically derived subset. Participants were not individually tracked between cycles; therefore, the cohorts represent independent cross-sectional samples rather than the same individuals.

Data collection procedures

Data were collected using a structured, self-administered questionnaire developed by the audit team in collaboration with the infection control committee and occupational health specialists. The questionnaire was designed in alignment with the WHO, CDC, and OSHA recommendations and underwent a pilot test for clarity and reliability prior to implementation [6,7,9]. It was divided into several sections. The first captured demographic data, including profession, department, and years of experience. The second focused on knowledge of sharps safety standards, such as awareness of the no-recap policy and availability of sharps containers at the point of care. A third section assessed understanding of immediate management steps following a needle-stick injury, while the fourth addressed awareness of the correct reporting procedures and timelines. The fifth section evaluated staff knowledge of hepatitis B vaccination status, availability, and initiation of HIV PEP, and appropriate follow-up investigations for blood-borne pathogens.

In the first audit cycle, the questionnaire was distributed across clinical departments to capture baseline knowledge levels. The responses were collated, analyzed, and used to identify key areas of deficiency in staff knowledge and awareness. After targeted interventions were implemented, the same questionnaire was re-administered during the second cycle to assess the impact of educational measures on staff knowledge and compliance with institutional and international standards.

Interventions between audit cycles

Several interventions were introduced following the first cycle in order to improve staff awareness and compliance. Structured teaching sessions were delivered by infection control staff and focused on essential topics such as safe sharps handling, adherence to the no-recap policy, and the correct post-exposure management steps to be followed in the event of a needle-stick injury. To reinforce these teachings, posters and visual reminders were prominently displayed in clinical areas. These materials summarized immediate first-aid procedures, outlined clear reporting channels, and highlighted the availability of PEP for HIV and hepatitis B.

Departmental meetings were also conducted, during which supervisors discussed sharps safety policies with staff and emphasized the importance of a supportive, non-punitive culture around incident reporting. This was intended to address the barrier of underreporting, which is well-documented in occupational health studies. In addition, printed guideline leaflets summarizing the hospital’s protocols for needle-stick injury management were distributed to staff across relevant departments, ensuring that healthcare workers had access to concise reference material at the point of care. These interventions were implemented progressively throughout March 2025, allowing for broad staff engagement and participation before the second cycle of the audit commenced.

Data analysis

Responses from the questionnaires in both audit cycles were coded, tabulated, and analyzed using MS Excel (Microsoft Corporation, Redmond, Washington, United States). Simple descriptive statistics were used to summarize staff knowledge levels, with results expressed as frequencies and percentages. No inferential statistical testing was conducted, as the aim of the audit was to describe changes in knowledge and awareness following educational interventions. The results were presented in tabular and graphical form to illustrate knowledge gaps, improvements achieved, and areas requiring further reinforcement. 

Ethical considerations

The audit protocol received approval from the hospital’s ethical review committee. All participants were informed of the audit’s purpose, and their participation was voluntary. Completion of the questionnaire was considered as implied consent. No personal identifiers were collected, and responses were anonymized during data entry and analysis. The results were reported in aggregate form to protect confidentiality. As no patients were directly involved in the study and data collection was limited to staff knowledge, the audit was classified as a quality improvement initiative and not as human subjects’ research. All ethical guidelines related to occupational health audits and quality improvement activities were strictly followed. 

## Results

Among doctors, several knowledge indicators improved across the audit cycles, particularly recognition of HBV as the primary pathogen, immediate first-aid responses, and awareness of PEP and its timing. However, awareness of HIV risk declined sharply, and familiarity with safety-engineered devices and correct sharps box disposal remained limited. Safe disposal without recapping deteriorated, and multiple training indicators declined, including formal training, confidence in training adequacy, and awareness of the reporting system. Full details are presented in Table 1.

**Table 1:** Changes in doctors’ knowledge, awareness, and training related to needle-stick injuries across two audit cycles This table presents findings from doctors only (Cycle 1: n = 50; Cycle 2: n = 50). Results are shown as frequencies with percentages in parentheses. The “change/analysis” column indicates the direction and magnitude of change between the two cycles. Separate results for nurses and laboratory technicians will be reported in subsequent tables

Category	Cycle 1 (n = 50)	Cycle 2 (n = 50)	Change/analysis
Most common pathogen identified as HBV	8 (16%)	41 (82%)	Major improvement (+66%)
Most common pathogen identified as HIV	34 (68%)	0 (0%)	Major decline (-68%)
Most common pathogen identified as HCV	8 (16%)	6 (12%)	Stable
Immediate response: wash with soap and water	37 (74%)	47 (94%)	Clear improvement (+20%)
Correct understanding of PEP as medication	26 (52%)	47 (94%)	Strong improvement (+42%)
Awareness PEP should be started within 24 hrs	24 (48%)	44 (88%)	Improvement (+40%)
Knowledge of safety-engineered devices	8 (16%)	13 (26%)	Minimal improvement (+10%)
Knowledge: sharps box to be stopped at 3/4 full	29 (58%)	34 (68%)	Slight improvement (+10%)
Dispose needles directly in sharps container without recapping	37 (74%)	19 (38%)	Decline (–36%)
Formal training received	37 (74%)	16 (32%)	Major decline (–42%)
Regular participation in refresher training	13 (26%)	13 (26%)	No change (0%)
Awareness of formal reporting system	45 (90%)	6 (12%)	Major decline (–78%)
Confidence in training adequacy	34 (68%)	25 (50%)	Decline (–18%)

Among laboratory technicians, knowledge and awareness improved substantially across most indicators, including recognition of HBV, immediate first-aid responses, PEP-related knowledge, disposal practices, and use of safety-engineered devices. Training coverage and confidence in training adequacy also increased. However, participation in refresher training declined, and awareness of the formal reporting system dropped sharply. Detailed findings are presented in Table 2.

**Table 2:** Changes in laboratory technicians’ knowledge, awareness, and training related to needle-stick injuries across two audit cycles Comparison of laboratory technicians’ knowledge and awareness regarding needle-stick injuries between the first (Cycle 1) and second (Cycle 2) audit cycles. Data are presented as frequencies with percentages in brackets. The “change/analysis” column indicates relative improvement or decline between cycles

Category	Cycle 1 (n = 20)	Cycle 2 (n = 20)	Change/analysis
Most common pathogen identified as HBV	13 (65%)	20 (100%)	Improvement (+35%)
Immediate response: wash with soap and water	12 (60%)	20 (100%)	Improvement (+40%)
Correct understanding of PEP as medication	5 (25%)	20 (100%)	Improvement (+75%)
Awareness PEP should be started within 24 hrs	0 (0%)	20 (100%)	Improvement (+100%)
Knowledge of safety-engineered devices	3 (15%)	20 (100%)	Improvement (+85%)
Knowledge: sharps box to be stopped at 3/4 full	11 (55%)	20 (100%)	Improvement (+45%)
Dispose needles directly in sharps container without recapping	1 (5%)	20 (100%)	Improvement (+95%)
Formal training received	12 (60%)	20 (100%)	Improvement (+40%)
Regular participation in refresher training	3 (15%)	1 (5%)	Decline (-10%)
Confidence in training adequacy	7 (35%)	16 (80%)	Improvement (+45%)
Awareness of formal reporting system	10 (50%)	1 (5%)	Decline (-45%)

Among laboratory technicians, suggestions focused on additional training and improved safety devices, with emerging support for a more supportive reporting culture in the re-audit cycle. These results are summarized in Table 3.

**Table 3:** Laboratory technicians’ suggestions to reduce NSIs (Cycle 1 vs Cycle 2, n = 20 each) Comparison of suggestions provided by laboratory technicians in the first (Cycle 1) and second (Cycle 2) audit cycles. Data are expressed as frequencies with percentages in brackets

Suggestion	Cycle 1 (n = 20)	Cycle 2 (n = 20)
More training	17 (85%)	10 (50%)
Better safety devices	2 (10%)	8 (40%)
Regular monitoring and feedback	1 (5%)	0 (0%)
Supportive reporting culture	0 (0%)	2 (10%)

## Discussion

This audit demonstrated important improvements in healthcare workers’ knowledge and awareness of NSI prevention and management following targeted interventions but also revealed concerning gaps that persisted or worsened in specific areas. Notably, recognition of HBV as the most common pathogen, knowledge of immediate wound care, awareness of the role of PEP, and understanding of several disposal-related parameters improved markedly across doctors, nurses, and laboratory technicians. However, the specific practice of disposing needles without recapping declined among doctors in the re-audit cycle. In addition, coverage of formal and refresher training declined, confidence in training adequacy was inconsistent, and awareness of formal reporting systems decreased substantially, particularly among doctors and technicians.

The observed improvements in core domains of knowledge are consistent with international recommendations. The WHO’s injection safety guidelines, the OSHA Bloodborne Pathogens Standard, and the CDC/USPHS guidelines emphasize immediate wound care, avoidance of needle recapping, timely initiation of PEP, and adherence to HBV vaccination policies [6,7,9]. Our findings suggest that educational interventions can enhance alignment with these standards, although institutional and systemic barriers may undermine sustainability.

Similar trends have been reported in other settings. A recent systematic review found that global NSI prevalence among healthcare workers remains high, but training and adoption of safety-engineered devices significantly reduce risk [11]. A cross-sectional study in Abha City reported that although most healthcare workers were aware of NSI guidelines, many continued to practice recapping, and more than half of the incidents went unreported [12]. Likewise, a study from Karachi reported a high prevalence of NSIs in public tertiary hospitals, with many exposures unreported and limited use of protective measures [13]. These studies reinforce the importance of continuous education, institutional oversight, and policy reinforcement. This aligns with our findings of knowledge improvements but persistent deficits in reporting.

The marked improvement in awareness of HBV risk and first-aid measures likely reflects the emphasis placed on these topics during training sessions and poster campaigns. By contrast, deterioration in training coverage and reporting awareness may reflect high staff turnover, competing institutional priorities, or insufficient dissemination of institutional policies. This decline in awareness of the reporting system itself may further exacerbate the widely documented issue of under-reporting, which is often driven by fear of blame, administrative burden, and skepticism regarding follow-up effectiveness [14].

The mixed outcomes observed in this audit mirror findings from broader international research. A global systematic review reported that NSIs remain prevalent across all regions, with the highest burden in low- and middle-income countries where under-reporting and limited access to safety-engineered devices are common [11]. In Sub-Saharan Africa, a multicenter study found that fewer than half of healthcare workers who sustained NSIs formally reported the incident, and only a minority received appropriate follow-up care [15]. These findings emphasize that the challenges identified in our setting are not unique but reflect systemic issues globally.

These results have important implications for occupational health programs. Improving knowledge alone is insufficient without supportive organizational structures. For example, although staff recognized the importance of wound washing and PEP, the absence of robust reporting systems and low refresher-training rates suggests underlying structural weaknesses. This highlights the need for structured, recurrent training, supportive reporting cultures, and consistent monitoring to ensure translation of knowledge into practice.

The strengths of this audit include its multiprofessional scope, the inclusion of three distinct healthcare worker groups, and repeated measurements across two cycles, allowing assessment of change over time. However, several limitations should be acknowledged. The study was conducted in a single institution, limiting generalizability. Knowledge was assessed through self-administered questionnaires, raising the possibility of response bias. This response bias may also have operated differently across cycles; staff aware of re-auditing may have provided socially desirable responses in Cycle 2, potentially inflating apparent improvements, whereas lower engagement or questionnaire fatigue may conversely have reduced accuracy among some participants. The direction of this effect is therefore uncertain. In addition, the decline in reported training coverage may reflect recall bias or variation in staff participation between cycles.

Future interventions should prioritize integrating NSI prevention into mandatory induction and annual refresher programs, ensuring access to safety-engineered devices, and establishing a supportive reporting culture that minimizes stigma and administrative barriers. Collaboration with national health authorities could facilitate standardization of monitoring, reporting, and follow-up systems. Overall, this audit demonstrates that targeted educational interventions can improve healthcare worker knowledge, but sustained institutional commitment is required to achieve lasting improvements in sharps safety. 

## Conclusions

This audit showed that targeted interventions improved healthcare workers’ knowledge of NSI prevention and management, particularly in wound care, PEP awareness, and HBV risk recognition. However, declines in training coverage and reporting awareness highlight the need for sustained institutional support, regular refresher programs, and a supportive reporting culture to achieve lasting improvements in sharps safety. While these interventions demonstrated short-term gains, they did not establish sustainable training or reporting infrastructure, underscoring the need for formal policy integration, real-time reporting pathways, and administrative oversight. In conclusion, structured educational programs should be embedded into routine hospital policy to ensure continuity, accountability, and long-term improvements in occupational safety, especially in resource-limited settings.

## References

[1] Abdelmalik MA, Alhowaymel FM, Fadlalmola H (2023). Global prevalence of needle stick injuries among nurses: a comprehensive systematic review and meta‐analysis. J Clin Nurs.

[2] World Health Organization (2003). Sharps Injuries: Global Burden of Disease From Sharps Injuries to Health-Care Workers. https://www.who.int/publications/i/item/9241562463.

[3] Kyle E (2024). Sharps safety. AORN J.

[4] (2025). Bloodborne pathogens. https://www.osha.gov/laws-regs/regulations/standardnumber/1910/1910.1030.

[5] (2022). Global, regional and national incidence and causes of needlestick injuries: a systematic review and meta-analysi. https://www.emro.who.int/emhj-volume-28-2022/volume-28-issue-3/global-regional-and-national-incidence-and-causes-of-needlestick-injuries-a-systematic-review-and-meta-analysi.html.

[6] (2012). Bloodborne pathogens and needlestick prevention. https://www.osha.gov/bloodborne-pathogens.

[7] World Health Organization (2010). WHO best practices for injections and related procedures toolkit. https://www.who.int/publications/i/item/who-best-practices-for-injections-and-related-procedures-toolkit.

[8] Kuhar DT, Henderson DK, Struble KA (2013). Updated US Public Health Service guidelines for the management of occupational exposures to human immunodeficiency virus and recommendations for postexposure prophylaxis. Infect Control Hosp Epidemiol.

[9] Moorman AC, de Perio MA, Goldschmidt R (2020). Testing and clinical management of health care personnel potentially exposed to hepatitis C virus-CDC Guidance, United States, 2020. MMWR Recomm Rep.

[10] Schillie S, Murphy TV, Sawyer M (2013). CDC guidance for evaluating health-care personnel for hepatitis B virus protection and for administering postexposure management. MMWR Recomm Rep.

[11] Bouya S, Balouchi A, Rafiemanesh H (2020). Global prevalence and device-related causes of needlestick injuries among healthcare workers: a systematic review and meta-analysis. Ann Glob Health.

[12] Alsabaani A, Alqahtani NS, Alqahtani SS (2022). Incidence, knowledge, attitude and practice toward needle stick injury among health care workers in Abha City, Saudi Arabia. Front Public Health.

[13] Aslam M, Taj T, Ali A, Mirza W, Ali H (2010). Needle stick injuries among health care workers of public sector tertiary care hospitals of Karachi. J Coll Physicians Surg Pak.

[14] Mbaisi EM, Ng'ang'a Z, Wanzala P, Omolo J (2013). Prevalence and factors associated with percutaneous injuries and splash exposures among health-care workers in a provincial hospital, Kenya, 2010. Pan Afr Med J.

[15] Zafar A, Habib F, Hadwani R, Ejaz M, Khowaja K, Khowaja R, Irfan S (2009). Impact of infection control activities on the rate of needle stick injuries at a tertiary care hospital of Pakistan over a period of six years: an observational study. BMC Infect Dis.

